# Safety Evaluation of Cervical Dorsal Instrumentation in Geriatric Patients: Experience at a Level 1 Center for Spinal Surgery—A Single Center Cohort Study

**DOI:** 10.3389/fmed.2022.824047

**Published:** 2022-05-18

**Authors:** Ehab Shabo, Simon Brandecker, Shaleen Rana, Gregor Bara, Jasmin E. Scorzin, Lars Eichhorn, Hartmut Vatter, Mohammed Banat

**Affiliations:** ^1^Department of Neurosurgery, University Hospital Bonn, Bonn, Germany; ^2^Department of Anesthesiology and Intensive Care Medicine, University Hospital Bonn, Bonn, Germany

**Keywords:** cervical dorsal instrumentation, geriatric patients, complication rate, comorbidities, CCI and CDG scores

## Abstract

**Objective:**

Dorsal instrumentation of the cervical spine is an established treatment in spine surgery. However, careful planning is required, particularly in elderly patients. This study evaluates early clinical outcomes in geriatric patients undergoing complex spine surgery.

**Methods:**

In this retrospective, single center cohort study, we included all geriatric patients (aged ≥65 years) who underwent dorsal instrumentation between January 2013 and December 2020. We analyzed postoperative complications and the 30-day in-hospital mortality rate. Furthermore, the Charlson comorbidity index (CCI) and Clavien-Dindo grading system (CDG) were used to assess the patients' comorbidity burden.

**Results:**

In total, 153 patients were identified and included. The mean age of patients was 78 years (*SD* ± 7). Traumatic injury (53.6%) was the most common reason for surgery. 60.8% of the patients underwent dorsal instrumentation with 3 or more levels. The most common comorbidities were arterial hypertension (64%), diabetes mellitus (22.2%), coronary heart disease and atrial fibrillation (19.6%). The most common adverse event (AE) was pneumonia (4%) and the most common surgery-related complication was wound infection (5.2%). Among patients categorized as high risk for AE (CCI > 5), 14.6% suffered a postoperative AE. In our univariate analysis, we found no risk factors for high rates of complications or mortality.

**Conclusion:**

Our data demonstrates that older patients were at no significant risk of postoperative complications. The CCI/CDG scores may identify patients at higher risk for adverse events after dorsal instrumentation, and these assessments should become an essential component of stratification in this older patient population.

## Introduction

Dorsal instrumentation and fixation of the spine is an established treatment option for a range of spinal pathologies including instability of the spine due to trauma or neoplasia, adult degenerative spine diseases, and infection (1).

Geriatric patients play an increasingly important role in the world of spine surgery as life expectancy and the number of older patients undergoing spinal instrumentation continue to increase worldwide, in particular instrumentation of the cervical spine regardless of the underlying causes (2–6). Furthermore, patients with spine procedures suffer more from complications compared to patients undergoing other types of surgery (e.g., cardiothoracic surgery) (7).

Several studies have described postoperative complications in cervical spine surgery, however with some limitations (8). Complication rates in geriatric patient cohorts are as high as 60% (9, 10). The goal of spinal surgery is to minimize neurological deficits and to improve the patient's quality of life; nevertheless, surgical treatment is associated with a high risk of complications in this group of vulnerable patients, especially in those populations with a high ASA score (11).

This study evaluates early clinical outcomes in geriatric patients undergoing complex spinal surgery at our center. The complication rates and 30 days in-hospital mortality were compared.

## Methods

### Patient Selection and Inclusion Criteria

In this retrospective single center cohort study, we analyzed all patients aged ≥65 years who underwent spine surgery by dorsal instrumentation and stabilization at our level 1 spine center between January 2013 and December 2020. The inclusion criteria were primary instability of the cervical spine after trauma, tumor, infection, or a degenerative spine disease such as ossification of the posterior longitudinal ligament (OPLL), amongst others.

We excluded all patients who were deemed not suitable for surgery and those with incomplete data and/or follow-up information.

Patient clinical information including age, sex, BMI, ASA score, associated comorbidities, operative duration, localization, postoperative complications, and 30 days in-hospital mortality were assessed. Furthermore, the Charlson comorbidity index (CCI) and Clavien-Dindo grading system (CDG) were applied to assess the patients' comorbidity burden (12, 13).

Surgery-related and in-hospital postoperative complications were defined as such adverse events occurring within 30 days of the initial surgery (7, 14).

Patients underwent standardized preoperative clinical and radiological (MRI and CT) examinations. A follow-up CT scan was routinely performed after surgery. Further clinical and imaging assessments were undertaken in the case of new or worse neurological deficits.

This study was performed in line with the ethical standards of our institutional and national research committee (Ethics committee of the Rheinische Friedrich Wilhelms University Bonn) and with the 1964 Helsinki declaration and its later amendments or comparable ethical standards. The local ethics committee at the University of Bonn approved this study (protocol no. 067/21).

### Surgical Procedure

The surgical procedure generally involved general anesthesia and a median dorsal approach. In case of dorsal fixation of the upper cervical vertebrae (C1-2) the Harms technique was used (15), whilst lateral mass screw fixation was used in the subaxial spine (16). A postoperative CT scan was conducted immediately after surgery. To reduce bias due to skill set or experience of the surgeon, all operations were conducted by four experienced neurosurgeons only.

### Radiological Evaluation

Postoperative imaging data was analyzed by an independent neuroradiologist in accordance with the institution's standards.

### Statistical Analysis

Data analysis was performed using IBM® SPSS® Statistics (version 25, IBM Corp., Armonk, NY). Quantitative, normally distributed data is presented as mean values ± standard deviation (SD), while non-parametric data is summarized by median values [first quartile–third quartile]. In the case of categorical variables, data is given as numbers and percentages. After normality testing via the Shapiro-Wilk test, continuous normally distributed data was compared using *t*-tests, while the Mann-Whitney *U* test was used for non-parametric data. Nominal data was tested between groups using Fisher's exact test and in the case of multinomial data with a chi-squared test. *P* value was calculated by dividing the standard *p*-value of 0.05 by all equally important variables. Thus in this case, the *p*-value was 0.05/11 = 0.004545, which was rounded to 0.005.

A *p* < 0.005 was considered statistically significant. About the chosen the *p*-value (0.005): statistically, the calculation of *p*-value when all variables are equally important (such as finding potential risk factors like this paper) is done by dividing the standard *p*-value (which is worldwide 0.05) by the number of the variables (17).

## Results

A total of 153 geriatric patients underwent cervical dorsal instrumentation between January 2013 and December 2020. Table 1 shows the baseline data. The mean age was 78 ± 7.12 (*SD*) years.

**Table 1:** Baseline data.

**Total (*N* = 153)**	**No. patients (%)**
**Age** (mean ± SD) (yrs.)	78 ± 7.12
**Sex**	
Male	93 (60.78)
Female	60 (39.21)
**BMI** (mean ± SD) kg/m^2^	25 ± 3.54
**ASA score**	
1&2	51 (33.33)
3&4	102 (66.66)
**Associated comorbidities**	
Arterial hypertension	98 (64.05)
Coronary heart disease	30 (19.6)
Atrial fibrillation	30 (19.6)
Diabetes mellitus	34 (22.22)
Kidney disease	22 (14.37)
History of PE/DVT/COPD	24 (15.68)
Heart insufficiency	7 (4.57)
Cardiomyopathy/Valve disease	18 (11.76)
**Length of stay in days** (mean ± SD)	16 ± 21.44
**Duration of operation in min**. [q1–q3]	213 [154–266]
**Instrumentation level**	
1. Level	41 (26.79)
2. Levels	19 (12.41)
3. Levels and more	93 (60.78)
**Localization**	
Upper cervical spine	65 (42.48)
Subaxial	88 (57.51)
**Pathology**	
Trauma	82 (53.59)
Degenerative	46 (30.06)
Tumor	15 (9.80)
Infection	10 (6.53)
**Postoperative complication**	
**No complications**	125 (81.7)
**Surgery-related complications:**	
Symptomatic vertebral artery injury	5 (3.26)
Wound infection	8 (5.22)
Hematoma	1 (0.65)
**Adverse events**	
Pneumonia	6 (3.92)
PE/DVT	3 (1.96)
Sepsis	2 (1.3)
Death (30 day mortality)	3 (1.96)
**Clavien-Dindo grading system (CDG)**	
Grade II	11 (7.18)
Grade III	9 (5.88)
Grade IV	5 (3.26)
Grade V	3 (1.96)

*ASA, American Society of Anesthesiology; BMI, Body Mass Index; COPD, chronic obstructive pulmonary disease; DVT, deep vein thrombosis; min., minutes; No., number; PE, pulmonary embolism; q1–q3, first quartile–third quartile; SD, standard deviation; yrs., years*.

Indications for instrumentation were classified into fractures due to trauma (53.6%), degenerative multisegmental spinal canal stenosis including OPLL (30.1%), metastatic spine tumors (9.8%), and infection with instability (6.5%).

At least one comorbidity was present in 82.4% of the patients. The most common associated comorbidities consisted of arterial hypertension (64.1%), diabetes mellitus (22.2%), coronary heart disease (19.6%), and atrial fibrillation (19.6%) (Table 1).

### Postoperative Complications

Postoperative complications within 30 days after surgery were classified into surgery-related complications and adverse events (Table 1).

Overall, 28 out of 153 patients (18.3%) suffered early postoperative complications, with 14 patients having surgery-related complications and 14 experiencing adverse events (Table 1). The most common surgery-related complication was wound infection (5.2%) and the most common adverse event was pneumonia (4%). One patient suffered from a new neurological deficit (paraparesis) due to postoperative hematoma, which was surgically removed. Another five patients suffered from vertebral artery injury, three of them with infarction of the cerebellum without need of revision. They were all acutely treated with heparin and later with aspirin. Table 2 shows the univariate analysis.

**Table 2:** Comparison of patients without postoperative complications vs. surgery-related complications and/or adverse events.

**Total (*N* = 153)**	**Pts. w/o complications**	**Pts. with surg.-rel. complications**	**Pts. with postoperative AEs**	**p1 (No compl. vs. surg.-rel.)**	**p2 (No compl. vs. AEs)**
**No. of patients**	125	14	14		
**Mean age (yrs.)** mean ± (*SD*)	77 ± 7.3	79.6 ± 5.9	80 ± 6.4	0.304	0.141
**Gender (F/M)**	50/75	5/9	5/9	1.00	1.00
**BMI (kg/m**^**2**^**)**, mean ± (*SD*)	24.9 ± 3.5	23.9 ± 3.6	24.4 ± 3.7	0.317	0.665
**ASA score**				0.557	0.548
1 or 2	42	6	3		
3 or 4	83	8	11		
**CCI score**				0.132	0.071
1 or 2	57	5	4		
>3	68	9	10		
**Comorbidities**				0.465	1.00
Arterial hypertension	83	7	8	0.247	0.557
Coronary heart disease	24	2	4	1.00	0.481
Atrial fibrillation	22	5	3	0.147	0.717
Diabetes mellitus	24	7	3	0.016	0.735
Kidney disease	13	6	3	0.004	0.205
History of PE/DVT/COPD	19	2	3	1.00	0.465
**Length of stay in days** median [q1–q3]	15 [9–30]	20 [13–51]	26 [8–41]	0.182	0.981
**Op duration in min.**, mean ± (SD)	212.3 ± 75.6	215.1 ± 57.8	217.9 ± 80.2	0.892	0.793
**OP Level**				0.035	0.115
1–2 level	55	1	4		
>3 levels	70	13	10		
**Localization**				0.262	0.092
Upper cervical spine	58	4	3		
Subaxial	67	10	11		
**Pathology**				0.227	0.077
Trauma	69	7	6		
Degenerative	38	5	3		
Tumor	13	0	2		
Infection	5	2	3		

*AEs, adverse events; ASA, American Society of Anesthesiology; BMI, body mass index; ds, days; CCI, Charlson comorbidity index; compl., complication; COPD, chronic obstructive pulmonary disease; DVT, deep vein thrombosis; PE, pulmonary embolism; pts., patients; q1-q3, first quartile – third quartile; surg.-rel., surgery-related; w/o, without; yrs., years*.

### Influence of Clinical Admission Status and CCI/CDG

All the patients suffering postoperative complications following dorsal cervical instrumentation exhibited similar values for ASA, BMI and age. There were no significant differences regarding outcome or postoperative complications in any of the patients. Among patients categorized as high risk for AE (CCI > 5), 14.6% suffered a postoperative AE. In our univariate analysis, we found no risk factors for high rates of complications or mortality (Table 2). Furthermore, we conducted subgroup analysis looking at the influence of age, comparing relatively older patients (aged over >80 yrs.) with younger patients. We found no significant effect in our patient cohort (*p* = 0.64).

### Influence of Patients' Comorbidities on Complications and Length of Hospital Stay

Patients with one or more of the comorbidities listed in Table 1 did not develop a significantly higher rate of postoperative complications and presented a similar outcome (as demonstrated by length of hospital stay) as patients without any comorbidities (Table 2).

### Influence of Operation Level, Duration of Operation, and Localization

Dorsal instrumentation took place at C1-2 level (Harms technique) in 42.5% of patients. Instrumentation for the other 57.5% of patients was subaxial (C3-7).

Operations were conducted in a single spinal level in 26.8% of patients, in two levels in 12.4% of patients, and in three or more levels in the remaining 60.8% of patients. The duration of the operation had no significant effect on complication rates.

This study shows no significant relationship between the operation level or localization and postoperative complications (Table 2).

### Influence of Pathology

We found no greater risk of complications depending on the indication for dorsal instrumentation, especially in the tumor or trauma subgroup.

## Discussion

Geriatric patients are an increasingly important group in medical care (18), while comorbidities require ever better planning before surgery (8, 19). As life expectancy worldwide continues to increase, the number of older patients undergoing cervical instrumentation is also increasing, regardless of the underlying causes (2–6, 10).

The present study evaluates the early outcome of dorsal instrumentation und fixation of the cervical spine in geriatric patients, focuses on postoperative complications in this specific cohort, and investigates the influence of comorbidities on complications.

The impact of spine surgery in geriatric patients is a subject of controversy in the literature (9, 10).

In one meta-analysis including 18 studies comparing elderly (*n* = 1,169) and non-elderly (*n* = 1,699) patients who received surgical treatment for cervical spondylotic myelopathy, no significant difference in the incidence of postoperative complications was noted. In addition, the complication rate in these geriatric patients was not significant (20). Razack et al. concluded that corrective procedures for symptomatic non-traumatic cervical myelopathy in elderly patients are safe and are not associated with significant postoperative complications (3). In another study, Kobayashi et al. concluded that preoperative motor deficits, operative time, estimated blood loss, and fusion surgery with instrumentation were significant risk factors for major complications. However, preoperative comorbidities were not significantly associated with postoperative complications (21). Kiyoshi et al. have shown that neither the cervical surgical procedure nor preoperative comorbidities had a significant effect on postoperative complications in elderly patients (22). Furthermore, several other publications do not show significantly higher postoperative complication rates between older and younger patients or in relation to preoperative comorbidities (2, 3, 20–28). However, most current studies discuss cervical procedures in geriatric patients with a non-traumatic cause.

Surgery on geriatric patients has some characteristics not present in the non-geriatric population, such as comorbidities, surgical treatment, or length of stay in hospital. As a result, one study recommends not treating every impairment (29). Generally, geriatric patients with relevant comorbidities suffered more complications after surgical treatment than younger patients (13, 30, 31). In the surgical and oncological world, CCI and CDG scores are widely used to predict complications, and are therefore applied in our study (13, 32–34). Timely identification of geriatric patients and peri-procedural classification of risk, combined with a clear therapy concept, have a positive influence on the outcome (35–38).

Fu et al. describe a high correlation between the ASA score of patients undergoing spinal surgery and their postoperative morbidity and mortality (11). Other studies correlate increased morbidity following spinal surgery with comorbidity factors such as age, ASA score, BMI or DM (39, 40).

In this study, we decided to investigate the relationship between patient condition/preoperative comorbidities and postoperative complications after dorsal instrumentation. Postoperative complications were divided into operation-related complications (symptomatic vertebral artery injury, wound infection, and hematoma) and adverse events (pneumonia, pulmonary embolism, sepsis, etc.). The cohort was defined as older patients of age ≥65 years suffering from pathologies (traumatic, degenerative, tumorous, and infectious) on whom we operated between January 2013 and December 2020. Our study shows that older patients were not at significant risk of developing postoperative complications, especially operation-related complications. Patients with postoperative adverse events were generally almost 4 years older than patients without any postoperative complications. This was not statistically significant (*p* = 0.14), however. This study shows that neither sex nor other aspects of the admission status of these patients (ASA score, BMI, etc.) had a significant influence on the complication rate after cervical spine surgery. The level and localization of the operation also seem to have had no significant effect on the overall outcome.

Our hypothesis was that there is a higher potential risk when operating on geriatric patients with comorbidities, but we found that this was not true, neither for operation-related complications nor for adverse events. Furthermore, withholding surgery from older patients could have a negative effect on their quality of life due to symptom progression.

Overall, this study clearly showed that comorbidities in our older patients were not associated with significantly higher complication rates and therefore did not influence the early outcome of cervical spine surgery.

## Conclusion

Since the overall population is clearly aging and there is a marked increase in average life expectancy, it is expected that the number of surgically treated geriatric patients will also increase in the future. It is therefore necessary to address this issue. Our data demonstrate that older patients were at no significant risk of postoperative complications. The CCI/CDG scores may identify patients at higher risk for adverse events after dorsal instrumentation and these assessments should become an essential component of stratification in this older patient population. Nonetheless, there is an associated predictor that is unlikely to be medical improved or changed. Early preoperative stratification of patients at risk may help to determine the optimal extent of postoperative monitoring and observation. This is useful for preoperative communication both with medical colleagues and with the affected patients and their families, as regards realistic needs and expectations of the therapy, especially of the neurosurgical treatment. Our recommendation meets the criteria for a level of evidence 3, based on the publication by Kaiser et al. (41).

## Limitations

The present study has several limitations. Data acquisition was retrospective. Furthermore, patients were not randomized, but treated according to the expert opinion of their neurosurgeon. Additionally, the present data represent only a single center experience.

## Data Availability Statement

The original contributions presented in the study are included in the article/supplementary material, further inquiries can be directed to the corresponding authors.

## Author Contributions

ES: conceived, designed and performed the study, first draft of the manuscript and illustrations, and analysis and interpretation of data. SB, SR, GB, JS, LE, and HV: critical review of the manuscript. MB: analysis, acquisition, interpretation of data, and supervision. The final manuscript was critically revised and approved by all authors.

## Conflict of Interest

The authors declare that the research was conducted in the absence of any commercial or financial relationships that could be construed as a potential conflict of interest.

## Publisher's Note

All claims expressed in this article are solely those of the authors and do not necessarily represent those of their affiliated organizations, or those of the publisher, the editors and the reviewers. Any product that may be evaluated in this article, or claim that may be made by its manufacturer, is not guaranteed or endorsed by the publisher.

## Abbreviations

AE: Adverse events
ASA: American Society of Anesthesiology
BMI: Body Mass Index
C1-7: Cervical vertebrae 1-7
CCI: Charlson comorbidity index
CDG: Clavien-Dindo grading system
CT: Computer tomogram
MRI: Magnetic resonance imaging
OR: Odds Ratio
OPLL: Ossification of the posterior longitudinal ligament
SD: Standard deviation.
